# Labor in the Hypnotic State

**Published:** 1886-05

**Authors:** 


### Labor in the Hypnotic State.

In the *Courier of Medicine*, a correspondent from Vienna reports a labor occurring to a woman, 26 years old, while under hypnotic influence.

In September last, it was found that she was very susceptible to hypnotic influence, by fixing her gaze upon any bright object held a short distance from the eyes.

On October 31st, in the morning, she came to the lying-in ward. Pains were frequent, prolonged, but weak. She continued in the first stage till 8 P. M., when the membranes were ruptured. The pains began to increase in force, and at 10.30 o'clock she was hypnotized by looking at the bright end of a thermometer. In a few seconds she became unconscious. In three minutes another pain of 30 seconds' duration came, and no evidence of her being conscious of it other than a slight twitching of the left hand as it began. The pains came regularly, increasing in force and duration till, at 11.15 P. M., she was delivered. She was unconscious of pain produced by pricking with a knife or pin, and the conjunctiva was insensible. She had the "bearing down," and it no doubt was reflex.

After delivery of the placenta, with some effort, she was partially aroused, and being asked how she felt said, "Very well, but very sleepy." She went to sleep again and did not awake till 4 A. M. She would not believe that she was delivered, was surprised at the smallness of her abdomen, and stated that she had experienced no pain.

